# Non-coding RNAs as Genetic Biomarkers for the Diagnosis, Prognosis, Radiosensitivity, and Histopathologic Grade of Meningioma

**DOI:** 10.7759/cureus.34593

**Published:** 2023-02-03

**Authors:** Akram M Eraky

**Affiliations:** 1 Neurological Surgery, Medical College of Wisconsin, Milwaukee, USA

**Keywords:** radiosensitivity, radiotherapy (rt), parasagittal meningioma, genetic biomarkers, meningioma, long non-coding rna, microrna, mirna, lncrna, non-coding rnas

## Abstract

Meningioma is considered the most common primary benign brain tumor. It originates from the arachnoid cells of the leptomeninges surrounding the brain. The mainstay treatment of meningiomas is microsurgical resection. Meningioma prognosis depends on tumor grade, location, and patient age. Recently, using non-coding RNA as a prognostic and diagnostic biomarker for many tumors became a trend. Herein, we demonstrate the importance of non-coding RNAs, including microRNAs and lncRNAs in meningioma and their potential role in meningioma's early diagnosis, prognosis, histological grade, and radiosensitivity. In this review, many microRNAs were found to be upregulated in radioresistant meningioma cells such as microRNA-221, microRNA-222, microRNA-4286, microRNA-4695-5p, microRNA-6732-5p, microRNA-6855-5p, microRNA-7977, microRNA-6765-3p, and microRNA-6787-5p. Moreover, there are many microRNAs downregulated in radioresistant meningioma cells such as microRNA-1275, microRNA-30c-1-3p, microRNA-4449, microRNA-4539, microRNA-4684-3p, microRNA-6129, and microRNA-6891-5p. Also, we highlight the possible use of non-coding RNAs as serum non-invasive biomarkers and their potential role as therapeutic targets to treat high-grade meningiomas. Recent studies show that microRNA-497, microRNA-195, microRNA-18a, microRNA-197, and microRNA-224 are downregulated in the serum of patients with meningiomas. Additionally, microRNA-106a-5p, microRNA-219-5p, microRNA-375, and microRNA-409-3p are found to be upregulated in the serum of patients with meningioma. We also found that there are many deregulated microRNAs in meningioma cells that can be used as potential biomarkers for meningioma diagnosis, prognosis, and histopathologic grade, such as microRNA-17-5p, microRNA-199a, microRNA-190a, microRNA-186-5p, microRNA155-5p, microRNA-22-3p, microRNA-24-3p, microRNA-26-5p, microRNA-27a-3p, microRNA-27b-3p, microRNA-96-5p, microRNA-146a-5p, microRNA-29c-3p, microRNA-219-5p, microRNA-335, microRNA-200a, microRNA-21, microRNA-107, microRNA-224, microRNA-195, microRNA-34a-3p, and microRNA-let-7d. Of interest, we found fewer studies discussing deregulated long non-coding RNAs (lncRNAs) in meningioma cells. LncRNAs work as competitive endogenous RNA (ceRNA) by binding to oncogenic or anti-oncogenic microRNAs. We found that lncRNA- NUP210, lncRNA-SPIRE2, lncRNA-SLC7A1, lncRNA-DMTN, lncRNA-LINC00702, and lncRNA-LINC00460 are upregulated in meningioma cells. In contrast, lncRNA-MALAT1 was found to be downregulated in meningioma cells.

## Introduction and background

Meningioma is considered the most common primary benign brain tumor. It originates from the arachnoid cells of the leptomeninges surrounding the brain [1]. The mainstay treatment of meningiomas is microsurgical resection. The meningioma prognosis depends on tumor grade, location, and patient’s age [1-4]. Chemotherapy and hormonal treatment have bad results in treating meningioma, despite meningiomas’ expression of hormonal receptors; subsequently, patients having recurrence after surgical resection and radiotherapy have very limited options for treatment [2,5-8]. As a result, searching for other potential therapies for meningioma should be encouraged by studying the role of non-coding RNAs on tumor differentiation, growth, and proliferation. According to the WHO classification, meningiomas are classified into three grades: classic (WHO-I), atypical (WHO-II), and malignant (WHO-III) [9]. If a meningioma meets three out of the following five histologic features, it is considered atypical meningioma. These features include small cells with a high nuclear: cytoplasmic ratio, prominent nucleoli, spontaneous necrosis, loss of whorling or fascicular architecture, and hypercellularity [10]. Less than 20% of meningioma cases are considered WHO-II or WHO-III and have aggressive behavior, high recurrence rate, and higher morbidity and mortality rates [2,4,9-11]. Recently, using non-coding RNA as a prognostic and diagnostic biomarker for many tumors became a trend [12]. Herein, we demonstrate the importance of non-coding RNAs’ expression in meningiomas and their potential role in meningioma’s early diagnosis. In contrast to the small part of our genome that is transcribed into messenger RNAs (mRNAs) to produce protein, most of our genome is transcribed into non-coding RNAs (ncRNA) that are not translated into proteins. Also, ncRNAs were considered to have unknown and less important roles than the protein-encoding genes [12]. Despite this, ncRNAs have been shown to have an important role in regulating gene expression, and cell differentiation [13]. NcRNAs having greater than 200 nucleotides (nt) are called long non-coding RNA (lncRNA) while those having 200 nt or less are considered small ncRNAs [12]. MicroRNAs are considered a type of small ncRNAs that regulates gene expression by binding to mRNAs [14]. It can bind to oncogenic or anti-oncogenic mRNAs; as a result, microRNAs may be able to suppress or induce tumor growth and proliferation [15]. In a large study containing 110 patients, Zhi et al. examined the expression of 200 microRNAs in meningioma cells and found that twelve microRNAs are significantly upregulated in meningioma cells and act as oncogenic factors [16]. These microRNAs include microRNA-17-5p, microRNA-199a, microRNA-190a, microRNA-186-5p, microRNA-155-5p, microRNA-22-3p, microRNA-24-3p, microRNA- 26b-5p, microRNA-27a-3p, microRNA-27b-3p, microRNA-96-5p, and microRNA-146a-5p [16]. They also found that microRNA-29c-3p and microRNA-219-5p are significantly downregulated in meningioma cells [16]. In this review, we focus on microRNAs that are significantly upregulated or downregulated in meningioma cells and are associated with meningioma cells’ proliferation, growth, and migration. LncRNAs can suppress or induce tumor growth and proliferation through many mechanisms. First, it can attach to the promoters of many tumor suppressor genes, enabling lncRNAs to silence those genes through its epigenetic modifications [12,17]. Second, it can bind to microRNAs and act as a competing endogenous RNA (ceRNA); as a result, it can alter microRNAs’ binding to protein-coding mRNAs [18]. Herein, we discuss dysregulated microRNAs and lncRNAs associated with meningioma growth, proliferation, and malignancy transformation.

## Review

MicroRNA-335 as a potential therapeutic target in meningioma and a possible biomarker of meningioma diagnosis

MicroRNA-335 is found to have a protective role in some tumors such as breast cancer metastasis, and malignant astrocytoma [19,20]. In contrast to the anti-oncogenic role of microRNA-335 in these tumors, Shi et al. found that microRNA-335 has an oncogenic effect in meningioma [11]. They showed that the inhibition of microRNA-335 induces cell arrest and suppresses cell proliferation. They suggested that this happened due to microRNA-335 ability to decrease the expression of the tumor-suppressing human retinoblastoma 1 (Rb1) protein. Moreover, they found that overexpression of microRNA-335 induces cell growth and proliferation [11]. This suggests the possibility of using microRNA-335 as a therapeutic target in meningioma treatment.

MicroRNA-200a as a potential therapeutic target in meningioma and a possible biomarker of meningioma prognosis

Senol et al. found that microRNA-200a inhibits meningioma cells’ growth and migration by decreasing the expression of the non-muscle heavy chain IIB (NMHCIIB) protein by targeting its mRNA [21]. They also found that on microRNA-200a overexpression, cells from malignant meningiomas (WHO-III) showed a significant decrease in migration [21]. Of interest, overexpression of microRNA-200a has been found to decrease the migration of tumor cells such as breast epithelial cells, and nasopharyngeal carcinoma [22,23]. This shows the potential role of microRNA-200a as a therapeutic target to decrease the aggressiveness of malignant meningiomas. Further, it shows the possibility of using microRNA-200a as a biomarker for meningioma’s aggressiveness.

MicroRNA-21 and microRNA-107 as potential biomarkers associated with changes in the histopathologic grades

Katar et al. found that increased expression of microRNA-21 and decreased expression of microRNA-107 are significantly associated with higher histopathologic grades [24]. Of interest, overexpression of microRNA-21 is associated with grade 3 and 4 gliomas, compared to grade 1 gliomas and normal brain tissues [25-27]. Barnabo et al. and Shi et al. found that there is a positive correlation between glioma grade and microRNA-21 expression [25,27]. Furthermore, Teplyuk et al. found that increased expression of microRNA-21 is associated with more advanced disease in glioblastoma multiforme (GBM) and metastatic brain tumors [26]. Regarding microRNA-107, Song et al. found that microRNA-107 expression is negatively correlated with renal cell carcinoma’s stage and size [28]. They also showed that decreased expression of microRNA-107 is associated with the incidence of metastasis [28]. Li et al. suggested that by targeting CKD8 in meningioma cells, microRNA-107 inhibits migration and proliferation [29].

MicroRNA-224 as a potential biomarker associated with changes in the histopathologic grades and a possible biomarker for meningioma diagnosis

Wang et al. found that there is a higher microRNA-224 expression in meningioma cells compared to normal cells [30]. They also found that microRNA-224 expression is positively correlated with the histopathologic grade [30]. They suggested that microRNA-224 induces meningioma’s growth and proliferation by targeting the early growth response 2 (ERG2) protein’s expression, which is a contributor to the apoptosis process [30]. Interestingly, microRNA-224 has been reported to be positively correlated to poor prognosis and aggressive behavior in many tumors such as liver, gastric, lung, and prostate cancers [31-33].

MicroRNAs as potential therapies inducing radiosensitivity of meningioma cells and possible biomarkers for radioresistance

Ionizing radiation’s therapeutic effect is achieved by its ability to cause DNA damage, which induces several repair signaling cascades [34]. Subsequently, it leads to P53 protein phosphorylation [34,35]. Phosphorylated P53 induces the expression of various genes including the Phosphatase and Tensin Homolog (PTEN) gene. As a result, it induces cell arrest and apoptosis [34-36]. This shows that the PTEN protein has an antioncogenic effect, and its expression can be induced by ionizing radiation. In contrast to the previously discussed antioncogenic effect of ionizing radiation, ionizing radiation can induce epithelial-mesenchymal transition (EMT) and cancer cells’ invasive and migratory properties [37-40]. As a result, ionizing radiation can induce meningioma cells’ invasiveness, recurrence, or malignant transformation. This paradoxical effect of ionizing radiation encourages more research to find potential radiosensitive-inducing agents. In meningioma cells, Zhang et al. found that decreasing microRNA-221 and microRNA-222 expression can enhance the apoptosis-inducing effect of ionizing radiation by increasing PTEN levels [41]. Regarding other tumors, recent studies show the effect of co-suppression of both microRNA-221 and microRNA-222 expression on inducing radiosensitivity [42,43]. Zhang et al. found that decreased expression of microRNA-221 and microRNA-222 induces radiosensitivity in gastric cancer and GBM cells by increasing the expression of the PTEN gene [42,43]. Furthermore, Khoshinani et al. showed that microRNA-222 regulates radiosensitivity by targeting PTEN in colorectal cancer cells [44]. Similarly, Xue et al. found that anti-microRNA-221 induces radiosensitivity in colorectal cancer cells by regulating the expression of the PTEN protein [45]. In a retrospective study, Zhang et al. compared both radiosensitive patients versus radioresistant patients. There was no significant difference in gender, age, peritumoral edema, Ki-67 index, tumor size, and tumor location between these two groups [46]. They found that in patients with radio-resistance, there are seven significantly upregulated microRNAs (microRNA-4286, microRNA-4695-5p, microRNA6732-5p, microRNA6855-5p, microRNA7977, microRNA-6765-3p, microRNA 6787-5p) and seven downregulated microRNAs (microRNA-1275, microRNA-30c-1-3p, microRNA4449, microRNA-4539, microRNA-4684-3p, microRNA-6129, microRNA-6891-5p) [46]. All microRNAs associated with increased radiosensitivity or radioresistance are summarized in Table 1. This highlights the possibility of using ncRNAs as a potential therapeutic target to increase meningioma cells' responsiveness to radiotherapy.

**Table 1:** MicroRNAs expression in radio-resistant meningioma cells

MicroRNA	Expression in radioresistant cells	Reference
MicroRNA-221	Upregulated	[41]
MicroRNA-222	Upregulated	[41]
MicroRNA-4286	Upregulated	[46]
MicroRNA-4695-5p	Upregulated	[46]
MicroRNA-6732-5p	Upregulated	[46]
MicroRNA-6855-5p	Upregulated	[46]
MicroRNA-7977	Upregulated	[46]
MicroRNA-6765-3p	Upregulated	[46]
MicroRNA-6787-5p	Upregulated	[46]
MicroRNA-1275	Downregulated	[46]
MicroRNA-30c-1-3p	Downregulated	[46]
MicroRNA4449	Downregulated	[46]
MicroRNA-4539	Downregulated	[46]
MicroRNA-4684-3p	Downregulated	[46]
MicroRNA-6129	Downregulated	[46]
MicroRNA-6891-5p	Downregulated	[46]

MicroRNA-195 as a malignant meningioma suppressor and a potential biomarker for meningioma histopathologic grade

Song et al. found that increased expression of microRNA-195 significantly decreased meningioma cells’ proliferation, invasion, and migration by targeting fatty acid synthase (FASN), which is found to be upregulated in high-grade meningioma compared to grade 1 meningioma cells [47]. This shows the possibility of using microRNA-195 as a biomarker for meningioma’s histopathologic grade. Of interest, microRNA-195 is found to be downregulated in many tumors such as non-small-cell lung cancer, and hepatocellular carcinoma [48,49]. Also, Mao et al. reported that osteosarcoma cell migration and invasion are suppressed by microRNA-195 [50]. Song et al. also found that there are many lncRNAs, such as NUP210, SPIRE2, SLC7A1, and DMTN, act as ceRNAs by sponging microRNA-195 and preventing it from binding to mRNA [47]. As a result, these lncRNAs are considered oncogenic by increasing FASN expression by targeting microRNA-195 [47].

MicroRNAs as serum non-invasive biomarkers for meningiomas

Tang et al. examined the levels of serum microRNA-185 in patients with meningiomas, gliomas, acoustic neuroma, and pituitary adenoma [51]. They found that the plasma level of microRNA-185 is only significantly changed in gliomas [51]. Another failed trial to find serum biomarkers for meningioma is reported by Wang et al. [52]. They found that serum microRNA-21, microRNA-128, and microRNA-342-3p are insignificantly altered in meningiomas [52]. Fortunately, Negroni et al. found that in patients with high-grade meningiomas, serum levels of microRNA-497 and microRNA-195 are lower than in those who do not have meningiomas [53]. This demonstrates the potential use of microRNA-497 and microRNA-195 as serum biomarkers for high-grade meningioma. Furthermore, Li et al. found that serum and cerebrospinal fluid (CSF) levels of microRNA-18a are significantly lower in patients with invasive meningioma than in healthy subjects [54]. They also found that only CSF levels of microRNA-18a are significantly lower in invasive meningioma than in patients with non-invasive meningioma [54]. In another study, Zhi et al. found that in patients with meningioma, microRNA-106a-5p, microRNA-219-5p, microRNA-375, and microRNA-409-3p are increased in the serum [55]. In contrast to this, they found that serum levels of microRNA-197 and microRNA-224 decreased in those patients [55]. This highlights the possibility of microRNAs as serum biomarkers for meningiomas. Of interest, the effect of inhibition of one of the microRNAs reported by Zhi et al. is studied by Hu et al. [56]. They found that inhibition of microRNA-197 in meningioma cells by Quercetin induces apoptosis and inhibits proliferation [56]. Quercetin’s effect on meningioma cells’ proliferation was reported in a previous study by Piantelli et al. [57]. However, the molecular mechanism was unknown. MicroRNAs that are considered potential serum non-invasive biomarkers for meningioma are summarized in Table 2. More clinical studies are encouraged to study the correlation between the serum levels of different ncRNAs and menenigeoma's diagnosis, prognosis, histopathologic grade, and radiosensitivity.

**Table 2:** Serum microRNAs expression in meningioma patients

MicroRNA	Expression	Reference
MicroRNA-497	Downregulated	[53]
MicroRNA-195	Downregulated	[53]
MicroRNA-18a	Downregulated	[54]
MicroRNA-106a-5p	Upregulated	[55]
MicroRNA-219-5p	Upregulated	[55]
MicroRNA-375	Upregulated	[55]
MicroRNA-409-3p	Upregulated	[55]
MicroRNA-197	Downregulated	[55]
MicroRNA-224	Downregulated	[55]

MicroRNA-34a-3p as a potential biomarker for meningioma diagnosis

Ludwig et al. found that microRNA-34a-3p is lower in grade II meningiomas compared to grade I meningiomas. They also suggested using microRNA-34a-3p as a biomarker that can differentiate higher-grade meningiomas [58]. In another study, Werner et al. found that microRNA-34a-3p inhibits apoptosis, proliferation, and invasiveness by targeting SMAD4, FRAT1, and BCL2 [59]. SMAD4 has an antioncogenic effect in the early stages of tumor development; however, it has an oncogenic effect in the late stages by stimulating angiogenesis and EMT [60]. Lower levels of microRNA-34a-3p increase SMAD4 expression and induce meningiomas’ growth, invasiveness, and proliferation. BCL2 has an antiapoptotic role. High levels of BCL2 are associated with increased recurrence in patients with benign meningioma. Moreover, in patients with atypical meningioma, BCL2 is found to be associated with a shorter time to recurrence [61,62]. Lower levels of microRNA-34a-3p increase BCL2 expression and induce meningiomas’ growth, invasiveness, and proliferation. Similarly, lower levels of microRNA-34a-3p increase FRAT1 expression and induce meningioma proliferation and invasiveness [59].

MicroRNA-let-7d as a potential biomarker for meningioma proliferation and invasion

MicroRNA-let-7d downregulation is found to be associated with poor survival in head and neck squamous cell carcinomas [63]. Furthermore, in another study by Su et al., they found that microRNA-let-7d can suppress growth and metastasis in renal cell carcinoma [64]. In meningioma cells, Li et al. found that high levels of microRNA-let-7d suppress proliferation, and stimulate apoptosis by targeting Astrocyte Elevated Gene-1 (AEG-1) [65]. AEG-1 is an oncogenic protein. Its inhibition induces apoptosis in prostate cancer and retinoblastoma [65,66]. In contrast, AEG-1 overexpression leads to cervical cancer progression [67]. Similarly, AEG-1 is overexpressed in meningioma cells [65]. All microRNAs mentioned in this review and associated with meningioma growth, proliferation, and invasion are summarized in Table 3.

**Table 3:** MicroRNAs associated with meningioma growth, proliferation, and invasion

MicroRNA	Expression in meningioma	References
MicroRNA-17-5p	Upregulated	[16]
MicroRNA-199a	Upregulated	[16]
MicroRNA-190a	Upregulated	[16]
MicroRNA-186-5p	Upregulated	[16]
MicroRNA155-5p	Upregulated	[16]
MicroRNA-22-3p	Upregulated	[16]
MicroRNA-24-3p	Upregulated	[16]
MicroRNA-26-5p	Upregulated	[16]
MicroRNA-27a-3p	Upregulated	[16]
MicroRNA-27b-3p	Upregulated	[16]
MicroRNA-96-5p	Upregulated	[16]
MicroRNA-146a-5p	Upregulated	[16]
MicroRNA-29c-3p	Downregulated	[16]
MicroRNA-219-5p	Downregulated	[16]
MicroRNA-335	Upregulated	[11]
MicroRNA-200a	Downregulated	[21]
MicroRNA-21	Upregulated	[24]
MicroRNA-107	Downregulated	[24]
MicroRNA-224	Upregulated	[30]
MicroRNA-195	Downregulated	[47]
MicroRNA-34a-3p	Downregulated	[58]
MicroRNA-let-7d	Downregulated	[65]

lncRNAs as biomarkers for meningioma diagnosis, prognosis, and histopathologic grade

In contrast to microRNAs, fewer lncRNAs associated with meningioma progression, growth, and invasion are studied. These lncRNAs act as a molecular sponge or ceRNA by binding to microRNAs. As a result, it can decrease or increase oncogenic or antioncogenic proteins [18]. Song et al. also found that there are many lncRNAs, such as NUP210, SPIRE2, SLC7A1, and DMTN, act as ceRNAs by sponging microRNA-195 and preventing it from binding to mRNA [47]. As a result, these lncRNAs are considered oncogenic by increasing FASN expression by targeting microRNA-195 [47]. Li et al. found that in malignant meningioma, lncRNA-LINC00702 upregulates ZEB1 by binding to microRNA-4652-3p [68]. Furthermore, Xing et al. found that lncRNA-LINC00460 induces meningioma metastasis and progression by binding to microRNA-539/MMP-9 [69]. Also, lncRNA-MALAT1 is found to act as a ceRNA by targeting microRNA-145, which is considered an oncogenic microRNA. Subsequently, lncRNA-MALAT1 overexpression can lead to reduced meningioma invasiveness [70]. All lncRNAs mentioned in this review and associated with meningioma growth, proliferation, and invasion are summarized in Table 4.

**Table 4:** lncRNAs expression in meningioma cells

LncRNA	Expression in meningioma	Reference
LncRNA- NUP210	Upregulated	[47]
LncRNA-SPIRE2	Upregulated	[47]
LncRNA-SLC7A1	Upregulated	[47]
LncRNA-DMTN	Upregulated	[47]
lncRNA-LINC00702	Upregulated	[68]
lncRNA-LINC00460	Upregulated	[69]
lncRNA-MALAT1	Downregulated	[70]

## Conclusions

Non-coding RNAs, including microRNAs and lncRNAs, are potential biomarkers for meningioma diagnosis, prognosis, aggressiveness, histopathologic grade, and radiosensitivity. More clinical studies with large samples are encouraged to examine serum, biopsy, and CSF levels of these non-coding RNAs' sensitivity and specificity.
